# The CH24H metabolite, 24HC, blocks viral entry by disrupting intracellular cholesterol homeostasis

**DOI:** 10.1016/j.redox.2023.102769

**Published:** 2023-05-31

**Authors:** Yueming Yuan, An Fang, Zongmei Wang, Zhihui Wang, Baokun Sui, Yunkai Zhu, Yuan Zhang, Caiqian Wang, Rong Zhang, Ming Zhou, Huanchun Chen, Zhen F. Fu, Ling Zhao

**Affiliations:** a State Key Laboratory of Agricultural Microbiology, Huazhong Agricultural University, Wuhan, 430070, China; b Hubei Hongshan Laboratory, Wuhan, 430070, China; c Key Laboratory of Preventive Veterinary Medicine of Hubei Province, College of Veterinary Medicine, Huazhong Agricultural University, Wuhan, 430070, China; d School of Basic Medical Sciences, Fudan University, Shanghai, 200433, China

**Keywords:** CH24H, 24HC, Neuroinvasive virus, Multivesicular body/late endosome, Cholesterol metabolism

## Abstract

Cholesterol-24-hydroxylase (CH24H or Cyp46a1) is a reticulum-associated membrane protein that plays an irreplaceable role in cholesterol metabolism in the brain and has been well-studied in several neuro-associated diseases in recent years. In the present study, we found that CH24H expression can be induced by several neuroinvasive viruses, including vesicular stomatitis virus (VSV), rabies virus (RABV), Semliki Forest virus (SFV) and murine hepatitis virus (MHV). The CH24H metabolite, 24-hydroxycholesterol (24HC), also shows competence in inhibiting the replication of multiple viruses, including severe acute respiratory syndrome coronavirus 2 (SARS-CoV-2). 24HC can increase the cholesterol concentration in multivesicular body (MVB)/late endosome (LE) by disrupting the interaction between OSBP and VAPA, resulting in viral particles being trapped in MVB/LE, ultimately compromising VSV and RABV entry into host cells. These findings provide the first evidence that brain cholesterol oxidation products may play a critical role in viral infection.

## Highlights

• CH24H is upregulated in a mouse brain-derived cell line upon infection with several types of neuroinvasive viruses.
• 24HC induces cholesterol accumulation in multivesicular bodies or late endosomes.
• The CH24H metabolite 24HC may limit rhabdovirus replication at the viral entry stage.

## Graphical abstract

**Figure:**
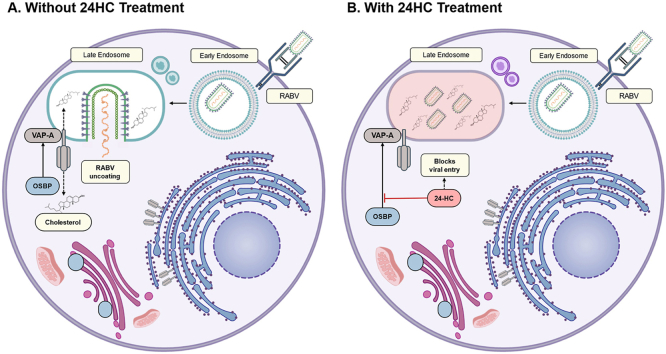

## 1 Introduction

Viruses that can infect the nervous system always pose great difficulties in terms of both treatment and prognosis. Due to the poor antiviral capacity of the neurological system, neuroinvasive viruses always present us with either high mortality or high recurrence rate [[1], [2], [3]]. Different neuroinvasive viruses can enter the brain through different cells depending on their different entry routes [4]. And infection with some types of neuroinvasive viruses can cause meningitis or encephalitis and lead to severe neurological dysfunction, such as rabies virus (RABV), vesicular stomatitis virus (VSV), Semliki Forest virus (SFV), and the culprit behind the recent global pandemic: severe acute respiratory syndrome coronavirus 2 (SARS-CoV-2) [[5], [6], [7], [8], [9]]. Therefore, there is an urgent need to develop new approaches for therapies as well as for more effective vaccines against the neuroinvasive virus.

The *Rhabdoviridae* family belongs to the *Mononegavirales* order. These enveloped virions are usually bullet shaped, 100–350 nm in length and 45–100 nm in diameter, and contain a single molecule of linear, negative single-stranded RNA. Their genome encodes the nucleoprotein (N), the phosphoprotein (P), the matrix protein (M), the glycoprotein (G), and the viral polymerase (L). The two most studied prototypes of the *Rhabdoviridae* family are VSV and RABV. During the adsorption process between VSV and RABV to the host cell membrane, the interaction of VSV-G and RABV-G with the specific cell surface receptors is usually required. It has been reported that the LDL receptor and its family members serve as the major entry port of VSV [10,11]. However, RABV uses receptors that are highly expressed on neurons, such as acetylcholine receptor and neural cell adhesion molecule [12,13]. After adsorption, VSV and RABV enter cells by clathrin-mediated endocytosis [[14], [15], [16], [17]]. The acidification of the interior of the endosome triggers fusion between the viral and the endosomal membranes, resulting in the release of viral RNP into the cytoplasm for infection to proceed.

Cyp46a1 (CH24H) is the P450 monooxygenase that plays an irreplaceable role in maintaining cholesterol homeostasis in the brain [18,19]. Recent studies have shown that CH24H is involved in many neurodegenerative diseases such as Alzheimer's disease (AD), Huntington's disease (HD), and multiple sclerosis (MS), as well as in brain disorders such as epilepsy [[20], [21], [22], [23]]**.** Animal studies have shown that the CH24H activity also affects cognitive and learning abilities [[24], [25], [26]]. Therefore, CH24H has emerged as a biomarker and a therapeutic target for neurodegenerative diseases and brain dysfunction [27,28].

24-Hydroxycholesterol (24HC) is converted from cholesterol by CH24H enzyme activity and can freely cross the blood-brain barrier (BBB), unlike cholesterol, which is BBB-impermeable [19,29]. Early studies have shown that the distribution of CH24H and 24HC is consistent and the amount is proportional [18]. As a metabolite of CH24H, the influence of 24HC on brain cholesterol metabolism and neuronal activity has also been investigated in recent years, showing that the concentration of 24HC is strongly associated with several types of brain disorders, such as epilepsy (SE) and bipolar disorder (BD) [29,30]. In addition, several *in vivo* studies have shown that the hyperphosphorylation of tau induced by amyloid β monomers can be completely prevented by intracerebroventricular injection of 24HC and that 24HC has beneficial modulatory effects on N-methyl-d-aspartate (NMDA) receptor (NMDAR) function, highlighting the neuroprotective role that 24HC may play in the brain [31,32].

It is widely accepted that many types of viral infection can alter host cholesterol metabolism such as infection with SARS-CoV-2 [33], rotavirus [34], or influenza A virus [35]. Cholesterol is required at different stages of the viral life cycle and inhibition of cholesterol biosynthesis pathways has been shown to reduce viral replication [[36], [37], [38], [39]]. In addition, modulation of cholesterol homeostasis within host cells, particularly in endosomes, has clear implications for the entry phase of viral infection [[40], [41], [42], [43], [44], [45], [46]]. Recently, an elegant study demonstrated that interferon-inducible transmembrane protein 3 (IFITM3) can block viral entry by disrupting intracellular cholesterol homeostasis [47], opening a whole new door to how host cells fight viral infection by using cellular cholesterol.

Here we report our discovery of the antiviral capacity of a natural brain cholesterol metabolite, 24HC. 24HC can inhibit the replication of several viruses, including two negative-strand RNA viruses: RABV and VSV, and three positive-strand RNA viruses: SFV, MHV, and SARS-CoV-2 *in vitro*. We discovered that the protein responsible for the production of 24HC, called CH24H, is mainly enriched in the cortex of the mouse brain and its mRNA level can be upregulated upon VSV infection. CH24H mRNA levels were significantly increased *in vitro* following infection with RABV, VSV, SFV, or MHV. Overexpression of CH24H could inhibit the replication of several viruses including RABV, VSV, SFV, and MHV. We further show that 24HC partially shares the same antiviral mechanism as 25HC or 27HC, which is the accumulation of cholesterol in MVBs/LEs by disrupting the OSBP-VAPA interaction. Thus, our study uncovers a natural antiviral small molecule present in the brain.

## 2 Materials and methods

### 2.1 Cell lines, viruses, antibodies, reagents, and mice

N2a (mouse neuroblastoma), BSR (cloned from baby hamster kidney-21, BHK-21), HEK-293T (human embryonic kidney, 293T), Vero (Cercopithecus aethiops kidney), L929 (mouse fibroblast) and MDCK (Madin-Daby canine kidney) cells were cultured in Dulbecco's modified Eagle's medium (DMEM) (Thermo Fisher Scientific, USA) supplemented with 10% (vol/vol) fetal bovine sera (FBS) (Thermo Fisher Scientific, USA) and 1% antibiotics (penicillin and streptomycin) (Beyotime, China). RABV CVS-B2c (B2c) strain (originated from the CVS-24 virus by passaging in BHK-21 cells) was stored in our lab. VSV and VSV-GFP are propagated in N2a cells and stored in our lab. SFV and MHV are both gifted from Dr. Bo Zhang (Wuhan Institute of Virology, Chinese Academy of Sciences, Wuhan, China), both of which are propagated in Vero cells.

The mouse monoclonal antibody (mAb) against RABV N or P protein, and the mouse polyclonal antibody (pAb) against VSV N protein were prepared by our lab. The mAbs against FLAG-tag (M185-3L), HA-tag (M180-3), and β-actin (M177-3) were purchased from Medical & Biological Laboratories (MBL, Nagoya, Japan). The pAbs against OSBP (11096-1-AP) and VAPA (15275-1-AP) were purchased from Proteintech (Chicago, USA). The Cyp46a1 (CH24H) pAb (A8573) was purchased from ABclonal Technology (Wuhan, China). The VSV-G Tag antibody (#81454), Rab7 rabbit mAb (#9367S), and c-Jun rabbit mAb (#9165) were purchased from Cell Signaling Technology (MA, USA).

The JNK inhibitor (#GC13841), the NF-κB inhibitor (#GC11751), the p38 inhibitor (#GC18602), the ERK inhibitor (#GC43624), 25-Hydroxycholesterol (25HC, #GC33860), 24-Hydroxycholesterol (24HC, #GC33673), Filipin III (#GC12048), Nile Red (#GC15539), and MβCD (#GC32697) were all purchased from Glpbio (GLPBIO, Montclair, USA).

All the experiments involving mice were performed following the recommendations in the Guide for the Care and Use of Laboratory Animals of the Ministry of Science and Technology of China and were approved by the Scientific Ethics Committee of Huazhong Agricultural University (permit number: HZAUMO-2022-0191).

### 2.2 Virus infection

N2a cells were infected with RABV, VSV, SFV, or MHV at a multiplicity of infection (MOI) of 0.01 or 10. BSR cells and 293T cells were infected with RABV or VSV at an MOI of 0.01. MDCK cells were used to infect with SARS-CoV-2 at an MOI of 0.01 or 0.001, respectively. After culturing at 37 °C with 5% CO_2_ for 1h, the supernatant was discarded and then the cells were washed three times with PBS. At last, the cells were added DMEM with 10% FBS and 1% antibiotics and cultured at 34 °C with 5% CO_2_ for indicated hours.

### 2.3 Virus titration

The procedure applied for RABV titration was determined by direct immunofluorescence assay using FITC-conjugated anti-RABV N antibody (Fujirebio Diagnostics, Malvern, PA) as described previously [48].

To determine VSV titers, BSR cells were infected with serial dilutions of the viruses. After 1 h incubation at 37 °C with 5% CO_2_, the cell supernatant was discarded and washed three times with PBS. After incubation at 34 °C with 5% CO_2_ for 24 h, the cells were probed with anti-VSV G antibodies (CST) and then treated with Alexa FluorTM 488 goat anti-rabbit IgG (A11034, Invitrogen) as secondary antibodies. The fluorescent foci were then counted under a fluorescence microscope.

Vero cells were used to determine SFV titers and L929 cells were used to determine MHV titers. Cells were seeded in 12-well plates and infected with serial dilutions of the viruses. After 1 h incubation at 37 °C with 5% CO_2_, the cell supernatant was discarded and washed three times with PBS and then supplied with DMEM containing 1% low melting point agarose. After a further incubation at 34 °C with 5% CO_2_ (48 h for SFV and 24 h for MHV), the agarose was removed and fixed. Then the fixed cells were stained with a solution of 0.1% crystal violet and 10% formalin in PBS under ultra-violet light. After staining for 4 h, the plates were washed and the plaques were counted.

### 2.4 Mouse infection

Female C57BL/6 mice (6 weeks old, n = 5) were infected intranasally with 20 μL of VSV (10 [5] FFU) or mock-infected intranasally with 20 μL DMEM. At 6 days post-infection (d.p.i.), mice were euthanized with CO_2_, and their brains were collected for RNA isolation and qPCR analysis.

### 2.5 Western blot

N2a cells or brain tissues were lysed in RIPA buffer (Beyotime, P0013) supplemented with 1 × protease inhibitor cocktail (Roche). The total cell lysates were separated on 12–14% SDS-PAGE gels and transferred to PVDF membranes (Bio-Rad). Membranes were blocked with TBST with 5% (w/v) non-fat dry milk for 4 h (h) and probed with primary antibodies which were diluted with TBST and 5% (w/v) non-fat dry milk overnight at 4 °C. After rinsing, membranes were probed with HRP-conjugated goat anti-mouse (BA1051, Boster, Wuhan, China), goat anti-rabbit secondary antibodies (BA1055, Boster), or goat anti-mouse IgG light-chain secondary antibodies (A25012, Abbkine, Wuhan, China), then developed using BeyoECL Star kit (P0018A, Beyotime). Images were captured with an Amersham Imager 600 (GE Healthcare) imaging system. ImageJ software (National Institutes of Health, Bethesda, MD, USA) was used to quantify the intensity of protein bands.

### 2.6 Quantitative real-time PCR (qPCR)

The brains and cells were subjected to RNA isolation using TRizol® reagent (Invitrogen) according to the manufactory's instructions, RNA quality was assessed by NanoDrop 2000 (Thermo Fisher Scientific) and the qPCR assays were performed as described previously 1. To quantify cellular RABV-N RNA levels, total RNA was transcribed using First-Strand cDNA Synthesis Kit (Toyobo, FSK-101) with a primer specific for the RABV-N gene. A standard curve was generated from serially diluted plasmids carrying the RABV-N gene and the copy number of N RNA was normalized to 1 μg of total RNA.

The corresponding primers used for qPCR as following: RABV-N-F: ACACCGCAACTACAAGACA; RABV-N-R: ATGGTACTCCAGTTGGCACA; CH24H–F:AGCCGCTATGAGCACATCC; CH24H–R: CCATACTTCTTAGCCCAATCCAG; β-actin-F: AGGTGACAGCATTGCTTCTG; β-actin-R: GCTGCCTCAACACCTCAAC.

### 2.7 Transfections

After seeding, cells were incubated for 12 h at 37 °C with 5% CO_2_. Plasmids or siRNA were transfected into cells by using jetPRIME® (Polyplus-transfection, France) according to the manufacturer's instructions.

### 2.8 siRNAs

The specific siRNAs targeting CH24H were designed and synthesized by GenePharma (Shang Hai, China). The negative control (NC) siRNA was purchased from GenePharma. The working concentration of 50 nM siRNAs was transfected into N2a cells according to the manufacturer's instructions. siCH24H–No.1 sequence was: 5′-GCAGCUGGUGGAAAUCCUATT-3’; siCH24H–No.2 sequence was: 5′-GCAUCAGUGCAUCCCGUAATT-3’; siCH24H–No.3 sequence was: 5′-GGAGGAGACCUUGAUUGAUTT-3’.

### 2.9 Cell viability assay

N2a cells were transfected with plasmids encoding CH24H for 48 h; N2a cells, BSR cells, 293T cells, and MDCK cells were treated with 24HC for 48 h. The viability of NA or MDCK cells was assessed using Cell Titer 96 AQueous One Solution cell proliferation assay kits (Promega, G3582) according to the manufacturer's instructions.

### 2.10 Construction of promoter-reporter plasmids

The CH24H promoter-reporter plasmids TSS-2500, TSS-2000, TSS-1500, TSS-1000, and TSS-500 contain the corresponding nucleotides proximal promoter sequences of CH24H were cloned by PCR amplification using genomic DNA of N2a cells as templates and subsequently cloned into PGL3-basic (Promega, Madison, WI). The CH24H promoter constructs containing site-specific mutation for the transcription factor binding site were constructed by overlap-expression PCR. All constructs were verified by sequencing.

The miR-200b-3p promoter constructs were amplified using the following primers. TSS-500 forward primer: 5′- GACGCTGGCACACAGCGGGC-3’ (chr12: 108300026–108300567); TSS-1000 forward primer: 5′- TCTTGTTCAGGCTGAGGTGGCCTCT-3’ (chr12: 108299526–108300567); TSS-1500 forward primer：5′- CCACTAAGCCAGATTGCTAACCCTT-3’ (chr12: 108299003–108300567); TSS-2000 forward primer: 5′- CAGCTTGTGTCGCACAGCTAACTATAGG-3’ (chr12: 108298526–108300567); TSS-2500 forward primer: 5′- AGTTGGAGCTCAGCAAGCCCATG-3’ (chr12: 108298026–108300567); Universal reverse primers： AACCACGTCTCCGCCTCCA.

### 2.11 Promoter activity assay

N2a cells were co-transfected with 100 ng of full-length or a series of truncated or mutant promoter firefly-luciferase reporter constructs and 10 ng of Renilla luciferase vector (pRL-TK). According to the manufacturer's protocol, luciferase activities were determined with the Dual-Luciferase Reporter Assay System (Promega) and expressed as relative luciferase activity by normalizing firefly luciferase activity against Renilla luciferase activity.

### 2.12 Chromatin immunoprecipitation (ChIP)

ChIP assay was performed according to the manufacturer's protocol for the ChromaFlash High-Sensitivity ChIP kit (Epigentek, Farmingdale, NY). Briefly, N2a cells were transfected with pCAGGS vector or pCAGGS-HA-c-Jun for 24 h, and the cells were infected with or without VSV. Then the growth media of N2a cells was removed, and cells were rinsed three times with cold PBS. Then cells were added with formaldehyde to a final concentration of 1% and incubated at room temperature for 15 min. Glycine was added to cells to a final concentration of 125 mM to stop the cross-linking reaction. The cells were then sonicated to a fragment size range of 100–700 bp. Immunoprecipitation was performed by incubating sheared chromatin overnight at 4 °C with HA-tag antibodies. The ChIP DNA was then extracted, and one-tenth of the purified sample was subjected to PCR amplification with primer pairs spanning transcription factor binding sites. PCR products were resolved by 2% agarose gel electrophoresis and visualized using UV light. The expression level of a target DNA sequence was determined relative to its abundance in the input chromatin.

### 2.13 ELISA

ELISA was performed to quantify the amount of 24HC in N2a cells. A commercially available mouse 24HC ELISA kit (#SP14965, Wuhan Saipei Biotechnology, China) was used following the manufacturer's instructions.

### 2.14 Confocal microscopy

N2a cells were seeded on 14-mm cell climbing slices. At different time points post-treatment, infection, or transfection, the cells were fixed with 4% paraformaldehyde for 30 min at room temperature and then washed three times with PBS. Then the cells were permeabilized with 0.1% Triton X-100, and then blocked with 10% goat serum (C0265, Beyotime) which were diluted with PBS for 2 h at 37 °C, and probed with antibodies against VSV-N (or RABV-N), Rab7 for 12 h at 4 °C. Then treated with Goat anti-mouse IgG DylightTM 594 conjugated antibodies (35511, Invitrogen) and Alexa FluorTM 488 goat anti-rabbit IgG (A11034, Invitrogen) as secondary antibodies for 1 h at room temperature. Nuclei were stained with 4, 6-diamidino-2-phenylindole (DAPI) (C1002, Beyotime) for 10 min at room temperature. Cells were again washed three times with PBS, and then imaged with a Zeiss LSM 880 confocal microscope under an oil objective (Carl Zeiss AG, Oberkochen, Germany). The resolution of 1024 × 1024 pixels, an average number of 2 pictures was utilized to acquire all confocal images.

For cholesterol staining, Filipin III (Glpbio) was used after fixing the cells. Results were imaged with ZEISS LSM880 confocal microscope under an oil objective (Carl Zeiss AG, Oberkochen, German).

To obtain the 3D images of transfected N2a cells, a resolution of 1024 × 1024 pixels, 1 μm Z-stack interval was utilized to acquire the desired volumes in cells, and the resulting z-stacks were imported into the ZEN software (version 2.3) to obtain a rendered 3D volume utilizing the acquisition parameters.

### 2.15 Flow cytometry

Flow cytometry was conducted to quantify cholesterol concentrations in 24HC- or 25HC-treated N2a cells. Briefly, N2a cells were stained with Nile Red and then collected and gone through a 40-mm nylon filter. The data collection and analysis were performed using a BD FACSVerse flow cytometer (BD Biosciences, CA, USA) and FlowJo software (TreeStar, CA, USA).

### 2.16 Co-immunoprecipitation

N2a cells with ethanol, 24HC, or 25HC treatment were washed with PBS lysed in RIPA buffer (P0013, Beyotime) supplemented with protease inhibitor cocktail (Roche) for 30 min on ice. The cell lysates were centrifuged for 10 min at 12,000 rpm and 4 °C, and the supernatants were transferred into a new tube and pretreated with rProtein A/G MagPoly Beads (SM01505, Smart-lifesciences, China) for 1 h at 4 °C to remove non-specific binding proteins from magnetic beads. Pretreated supernatants were further incubated with mAbs against OSBP or FLAG overnight at 4 °C, and then fresh magnetic beads washed with PBS were added and incubated for 3 h at 4 °C with rotation. The samples were then washed five times with ice-cold PBS, and the bound proteins were eluted by boiling in 4 × SDS-PAGE loading buffer and analyzed by Western blot with the indicated antibodies.

### 2.17 Statistical analysis

Data are expressed as the mean and standard deviation (SD), and the significance of the differences between groups was evaluated by Student's t-test followed by Tukey's post hoc test. The asterisks indicated statistical significance (*, P < 0.05; **, P < 0.01; ***, P < 0.001). Graphs were plotted and analyzed using GraphPad Prism software, version 8.0 (GraphPad Software, La Jolla, CA, USA).

## 3 Results

### 3.1 CH24H expression is upregulated following VSV infection and is regulated via MAPK pathways

**Fig. 1:**
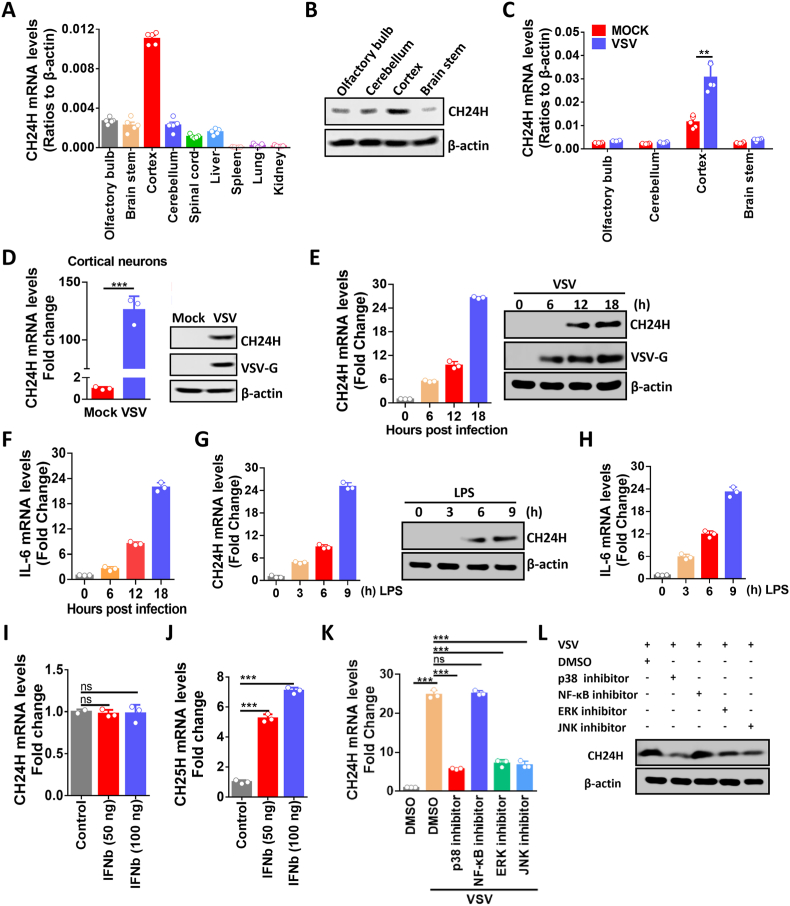
CH24H is enriched in the mouse cortex and is upregulated via MAPK pathways upon VSV infection *in vitro*. A. Basal levels of CH24H mRNA in different tissues of C57/BL6 mice (n = 5) were analyzed by qPCR. B. The basal level of CH24H protein in different areas of the brain was analyzed by Western blotting (WB), and β-actin was included as a reference gene. C. C57BL/6 mice (n = 5) were infected intranasally (i.n.) with VSV at 10^5^ FFU or mock infected with DMEM (Mock). At 6 days post-infection (d.p.i.), mouse brains were harvested and qPCR was performed to detect CH24H mRNA levels. D. Mouse cortical neurons were isolated and infected with VSV at MOI 0.01, cells were harvested at 24 h post-infection (h.p.i.), and CH24H mRNA and protein levels were measured by qPCR (n = 3) and WB. E, F. N2a cells were infected with VSV at MOI 0.01 and cells were harvested at the indicated time points. CH24H mRNA and protein levels (E), and IL-6 mRNA levels (F) were measured by qPCR (n = 3) and WB. G, H. N2a cells were incubated with LPS (100 ng/ml) for the indicated periods. CH24H mRNA and protein levels (G) and IL-6 mRNA levels (H) were measured by qPCR (n = 3) and WB. I, J. N2a cells were incubated with IFNβ at different concentrations (50 ng/ml and 100 ng/ml) for 24 h (h), and CH24H (I) and CH25H (J) mRNA levels were measured by qPCR (n = 3). K. N2a cells were mock infected or infected with VSV at an MOI of 0.01, at different time points after VSV infection, the culture medium was replaced in the presence of specific inhibitors (p38 inhibitor, 1 μM, 24 h; NF-κB inhibitor, 5 μM，18 h; JNK inhibitor, 0.5 μM, 12 h; ERK inhibitor, 5 μM, 18 h) or DMSO. CH24H mRNA levels were measured by qPCR (n = 3). L. N2a cells were infected with VSV at an MOI of 0.01, at different time points after VSV infection, the culture medium was replaced in the presence of specific inhibitors (p38 inhibitor, 1 μM, 24 h; NF-κB inhibitor, 5 μM，18 h; JNK inhibitor, 0.5 μM, 12 h; ERK inhibitor, 5 μM, 18 h) or DMSO. CH24H protein levels were measured by WB. Statistical analysis of comparisons between groups was performed by Student's t-test (*, P < 0.05; **, P < 0.01; ***, P < 0.001; ns, not significant). Bar graph shows mean ± SD. The Western blot data are representative of at least two independent experiments.

To investigate the role of CH24H in viral infection, we first examined the abundance of CH24H in various organs in the absence of viral infection. C57/BL6 mice were euthanized and their organs were harvested to detect the mRNA level of CH24H by quantitative real-time PCR (qPCR). The result showed that CH24H is mainly enriched in the cortex (Fig. 1A). Western blotting was used to confirm that the cortex has the highest CH24H protein level (Fig. 1B). Then, to investigate the change in CH24H abundance after viral infection, VSV was injected intranasally (i.n.) into mice and their brains were collected at 6 days post-infection (d.p.i.). qPCR results showed that CH24H mRNA level was significantly upregulated in the cortex after VSV infection (Fig. 1C). Since CH24H is enriched in neurons [18], we then isolated mouse cortical neurons and infected the cells with VSV, and the qPCR and Western blotting results were consistent with those obtained in VSV-infected mouse brains (Fig. 1D).

To further explain how CH24H expression was regulated, we chose the neuroblastoma cell line (N2a cells) for follow-up experiments. As shown in Fig. 1E, CH24H mRNA and protein levels were significantly upregulated upon VSV infection, and we also found that IL-6 mRNA levels were significantly upregulated upon VSV infection, suggesting that VSV infection may induce inflammation of N2a cells (Fig. 1F). CH24H expression has been reported to be closely associated with inflammation [49]. Also, VSV infection can induce a strong inflammatory response [50], so we wonder whether the inflammation induced by VSV infection mediates CH24H production.

LPS was then used to treat N2a cells for the indicated periods and it was found that both CH24H mRNA and protein levels were upregulated (Fig. 1G), with IL-6 as a positive control (Fig. 1H). Since CH24H and cholesterol-25-hydroxylase (CH25H) belong to the p450 enzyme family and CH25H has been confirmed as an interferon-stimulated gene (ISG) [51], we also tested whether CH24H is an ISG. The results showed that CH24H couldn't be upregulated under IFN-β treatment, whereas CH25H could (Fig. 1I and J).

Previous studies have shown that two primary inflammatory pathways, the NF-κB and MAPK pathways, are activated by VSV infection [50]. Signaling pathways mediated by the MAPK family, including extracellular signal-regulated kinases 1 and 2 (ERK1/2), c-Jun N-terminal kinase (JNK), and p38, contribute to the activation of transcription factors [52]. To further investigate which inflammatory pathway is responsible for CH24H production, VSV-infected N2a cells were treated with different inhibitors of the NF-κB (QNZ), p38 (p38 MAPK inhibitor IV), ERK (ERK inhibitor) and JNK (JNK–IN–8) pathways. The qPCR (Fig. 1K) and Western blot (Fig. 1L) results showed that the p38, ERK, and JNK pathways were associated with CH24H production, and the inhibition of the single pathway could not lead to the complete obliteration of CH24H production. Taken together, these data demonstrate that CH24H expression levels can be upregulated during VSV infection via MAPK pathways.

### 3.2 The transcription factor c-Jun binds to the CH24H promoter and regulates its activity

**Fig. 2:**
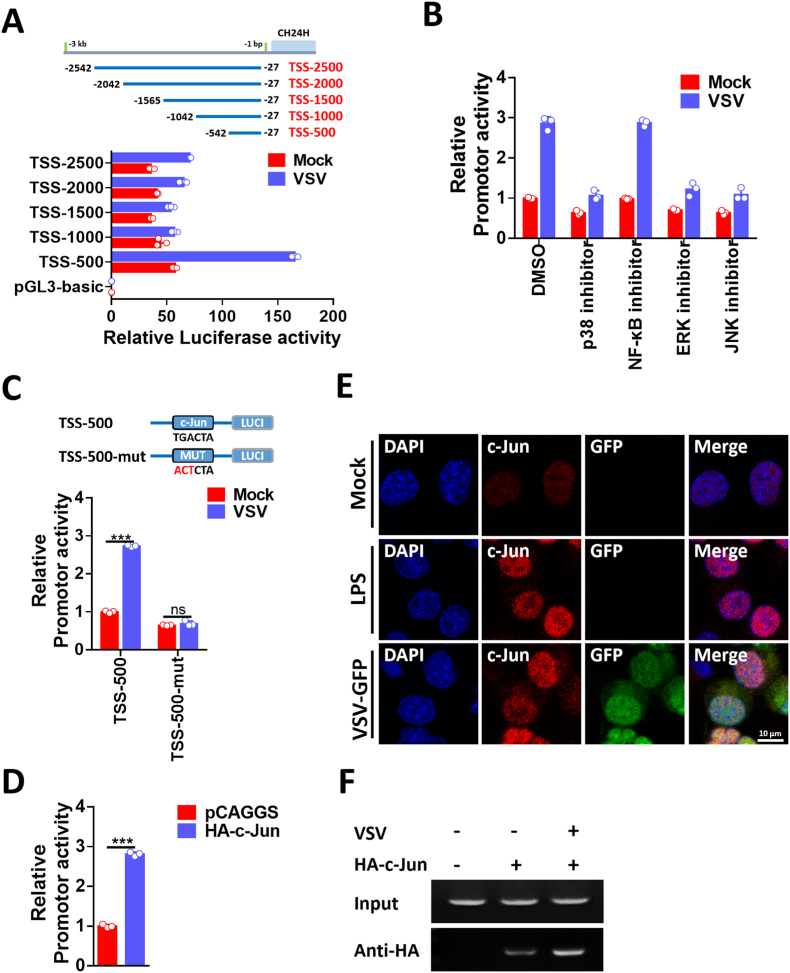
The transcription factor c-Jun element regulates the activity of the CH24H promoter. A. N2a cells were transfected with CH24H promoter-reporter plasmids (TSS-500, TSS-1000, TSS-1500, TSS-2000, and TSS-2500) for 24 h. Then the cells were either infected with VSV at an MOI of 0.01 or uninfected, and the luciferase activities were measured after another 18 h of infection (n = 3). B. N2a cells were transfected with the CH24H promoter-reporter plasmid: TSS-500. At 24 h post-transfection, cells were infected with VSV at an MOI of 0.01. At different time points after VSV infection, the culture medium was replaced in the presence of different signaling pathway-specific inhibitors (p38 inhibitor, 1 μM, 24 h; NF-κB inhibitor, 5 μM, 18 h; JNK inhibitor, 0.5 μM, 12 h; ERK inhibitor, 5 μM, 18 h) or DMSO. After 18 h of infection, the luciferase activity of the CH24H promoter was measured (n = 3). C. Schematic representation of point mutations in the wild-type promoter-reporter (TSS-500). N2a cells were transfected with the CH24H promoter reporter (TSS-500 and its mutant: TSS-500-mut). At 24 h post-transfection, cells were either mock-infected or infected with VSV at MOI 0.01, and luciferase activity was measured after 18 h of infection (n = 3). D. N2a cells were co-transfected with CH24H promoter-reporter (TSS-500) plasmid and pCAGSS-HA-c-Jun expressing plasmid (named HA-c-Fos) or pCAGGS vector for 24 h, and then CH24H promoter luciferase activity was measured (n = 3). E. N2a cells were mock-infected (top) or infected with VSV-GFP (bottom) at an MOI of 0.01. At 18 h post-infection, the cells were fixed, and c-Jun proteins (left), GFP (middle), and cell nuclei (right) were observed by confocal microscopy. Scale bar = 10 μm. F. N2a cells were transfected with HA-c-Jun for 24 h and then infected with VSV at an MOI of 0.01 for 18 h. Fixed chromatin from N2a cells was prepared and immunoprecipitated with anti-HA antibodies. ChIP primers were designed to amplify the region containing the c-Jun binding site in the CH24H promoter. PCR products were separated by acrylamide gel electrophoresis. Statistical analysis of comparisons between groups was performed by Student's t-test (*, P < 0.05; **, P < 0.01; ***, P < 0.001). Bar graph shows mean ± SD.

To further elucidate how CH24H expression is regulated, we analyzed the promoter region of CH24H. An approximately 2.5-kb DNA fragment containing non-coding sequences upstream of the CH24H transcriptional initiation site (TSS) was cloned for promoter mapping. Luciferase reporter genes with different lengths of the CH24H putative promoter regions were constructed and transfected into N2a cells. The basal and VSV-induced promoter activities were then evaluated. As shown in Fig. 2A, among the five reporters, the reporter TSS-500 had the highest luciferase activity in both mock-infected N2a cells and VSV-infected N2a cells, suggesting that TSS-500 had fully intact promoter activity. Therefore, the TSS-500 reporter was a CH24H promoter responsive to VSV infection and was selected for further analysis. To further analyze whether inflammatory pathways play an important role in the regulation of VSV-induced CH24H expression, specific inhibitors of signaling pathways were used. The results showed that the MAPK pathway was involved in the activation of the CH24H promoter (Fig. 2B).

Next, we used PROMO and JASPAR software to predict potential transcription factor-binding sites (TFBS) in the CH24H promoter region (TSS-500). The results showed that only one potential transcription factor, c-Jun, was found within the CH24H promoter. To determine whether c-Jun is responsive to CH24H transcription, the c-Jun binding site mutated promoter construct (TSS-500-mut) was generated and tested for its promoter activity under VSV infection. The results showed that compared with TSS-500, mutation of c-Jun binding sites could completely abolish VSV-mediated induction of CH24H promoter activity (Fig. 2C), suggesting that activation of the CH24H promoter by VSV infection requires c-Jun. We also constructed the expression plasmid pCAGGS-HA-c-Jun (named HA-c-Jun) and confirmed that the of HA-c-Jun could activate CH24H promoter activity (Fig. 2D).

Since transcription factors need to be transported into the cell nucleus and bind to appropriate DNA fragments to exert their function, we tested whether VSV infection could lead to the nuclear translocation of c-Jun. Our confocal microscopy analysis showed that c-Jun signals were weakly present in the nucleus of mock-infected cells, whereas c-Jun signals were significantly increased in the nucleus after VSV infection or LPS treatment (Fig. 2E). Furthermore, we used chromatin immunoprecipitation assay (ChIP) and confirmed that HA-c-Jun could bind to the promoter of CH24H after VSV infection (Fig. 2F). Taken together, these results demonstrate that the activity of the CH24H promoter is regulated by the transcription factor c-Jun.

### 3.3 Both CH24H and its metabolite 24HC limit viral replication

**Fig. 3:**
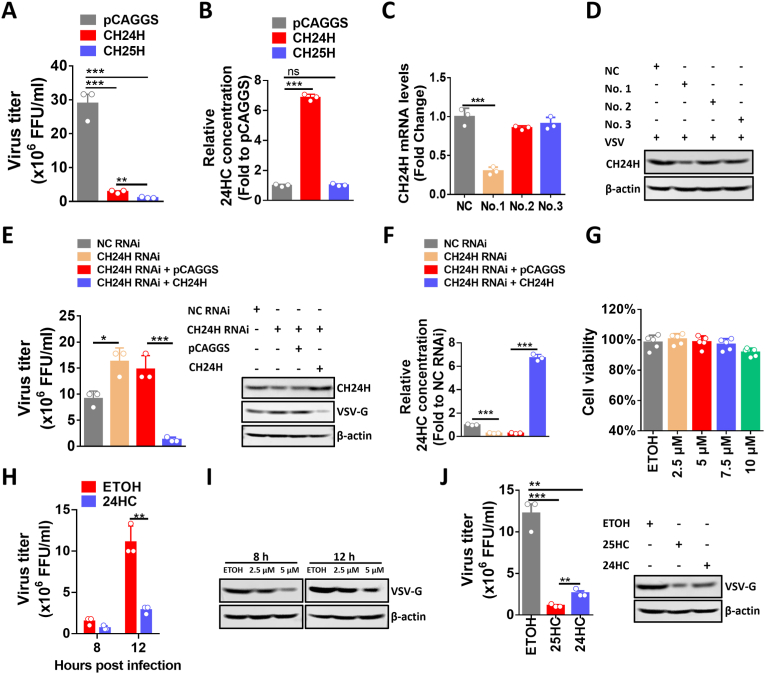
CH24H and 24HC inhibit VSV replication. A. N2a cells were transfected with the expression plasmid pCAGGS vector, pCAGGS-CH24H (named CH24H), or pCAGGS-CH25H (named CH25H), then the cells were infected with VSV at MOI 0.01 for 18 h, and viral titers were measured (n = 3). B. N2a cells were transfected as in (A), and then cells were lysed for 24HC concentration analysis by ELISA (n = 3). C. N2a cells were transfected with three different sets of CH24H-specific siRNAs (No.1, No.2, No.3) or negative control (NC), and CH24H mRNA levels were measured by qPCR at 36 h post-transfection (n = 3). D. N2a cells were transfected with three different sets of CH24H-specific siRNAs (No.1, No.2, No.3) or NC for 24 h, and then infected with VSV at MOI 0.01, CH24H protein levels were measured by WB at 18 h post-infection (h.p.i.) (n = 3). E. N2a cells were transfected with negative control siRNA (NC RNAi), CH24H-specific siRNA (CH24H RNAi), pCAGGS vector with CH24H RNAi, or CH24H with CH24H RNAi. Cells were infected with VSV at MOI 0.01 at 24 h post-transfection. After 12 h of VSV infection, viral titration (n = 3) and WB were used to measure viral load. F. N2a cells were transfected as in (C), and then cells were lysed for 24HC concentration analysis by ELISA (n = 3). G. N2a cells were treated with different concentrations of 24HC or ethanol (ETOH) for 36 h, then the supernatants were discarded and the cytotoxicity was measured (n = 5). H. N2a cells were pretreated with 5 μM 24HC for 12 h, and then infected with VSV at MOI 0.01 for the indicated periods, then supernatants were collected and viral titers were measured (n = 3). I. N2a cells were pretreated with 2.5 μM 24HC or 5 μM 24HC for 12 h, and then infected with VSV at MOI 0.01 for the indicated periods, WB was used to measure viral loads. J. N2a cells were pretreated with 5 μmol (μM) 24HC or 5 μM 25HC for 12 h, and then infected with VSV at MOI 0.01 for 12 h, viral titration (n = 3) (D) and WB (E) were used to measure viral loads. Statistical analysis of comparisons between groups was performed by Student's t-test (*, P < 0.05; **, P < 0.01; ***, P < 0.001; ns, not significant). Bar graph shows mean ± SD. Western blot data are representative of at least two independent experiments.

Having clarified how VSV infection upregulates CH24H expression, we sought to investigate the role of CH24H in VSV replication. Murine CH24H was cloned and inserted into the mammalian expression vector pCAGGS, resulting in pCAGGS-CH24H (named CH24H). Next, N2a cells were transfected with CH24H or CH25H (CH25H was added as a positive control according to our previous research [53]), and then their anti-VSV capacities were measured. The viral titration results showed that the overexpression of CH24H or CH25H could inhibit VSV replication, and CH25H showed stronger antiviral capacity compared with CH24H (Fig. 3A). Previous studies demonstrated that CH25H exerts its antiviral activity mainly through the production of 25-hydroxycholesterol (25HC) [54]. To measure the concentration of 24-hydroxycholesterol (24HC) produced by CH24H, ELISA was then used to detect the 24HC concentrations inside N2a cells under the overexpression of CH24H or CH25H, the results showed that CH24H overexpression could significantly upregulate 24HC levels, but CH25H overexpression could not (Fig. 3B).

To further confirm that CH24H can inhibit viral replication, RNA interference (RNAi) was used to knock down CH24H levels in N2a cells. Based on the RNAi efficiency, the No. 1 RNAi (named CH24H RNAi in follow-up experiments) of 3 pairs of RNAi primers was selected for further experiments (Fig. 3C and D). Next, CH24H siRNA was transfected into N2a cells and then infected with VSV, and the viral titration results showed that transfection of CH24H knockdown could increase the VSV replication level, and CH24H overexpression could reverse this trend (Fig. 3E). ELISA was also used to detect 24HC levels inside N2a cells when CH24H was knocked down, and the results showed that CH24H knockdown could significantly decrease 24HC levels inside cells, and CH24H overexpression could reverse this trend (Fig. 3F). These results suggest that CH24H influences VSV replication, and the level of 24HC may be related to the antiviral capacity of CH24H. To determine whether the antiviral effect of CH24H was cell-specific, we also tested the antiviral effect of CH24H on 293T cells. The viral titer and Western blot results showed that the replication of VSV was significantly decreased under the overexpression of human CH24H (Figs. S1A and S1B), suggesting that the antiviral effect of CH24H is not cell-specific.

To test whether 24HC could exert anti-VSV capacity, the MTT assay was performed to evaluate the cytotoxicity of synthetic 24HC on N2a cells. The result showed that no cytotoxicity could be detected when 5 μmol (μM) 24HC was added to N2a cell culture media for 36 h (Fig. 3G). Based on the cytotoxicity result, N2a cells were pretreated with 24HC for 12 h and then infected with VSV for the indicated periods. Viral titer and Western blot results showed that the replication of VSV was significantly decreased after treatment with 24HC (Fig. 3H and I). We also measured the degree of inhibition of VSV replication by 25HC treatment and found that 25HC had a stronger antiviral capacity compared to 24HC (Fig. 3J).

To determine whether the antiviral effect of 24HC is cell-specific, we tested the antiviral effect of 24HC on BSR and 293T cells. MTT assays were performed to evaluate the cytotoxicity of synthetic 24HC on BSR and 293T cells. The results showed that no cytotoxicity could be detected when 5 μM 24HC was added to BSR or 293T cell culture media for 36 h (Figs. S2A and S2B). Based on the cytotoxicity results, BSR and 293T cells were pretreated with 24HC for 12 h and then infected with VSV for the indicated periods. Viral titer and Western blot results showed that the replication of VSV was significantly decreased after treatment with 24HC (Figs. S2C–S2F), suggesting that the antiviral effect of 24HC is not cell-specific. Taken together, these results confirm the antiviral capacity of 24HC against VSV infection.

### 3.4 24HC limits viral replication at the viral entry stage

We then sought to determine which step of the early viral replication cycle was impaired by 24HC. The early stages of viral replication generally include contact between the virus and the cell membrane, virus internalization, and the release of the virus particles into the cytoplasm. The inactivation assay was used to test whether 24HC is virucidal and thus kills the virus. The adsorption assay was used to test whether 24HC interferes with virus contact with cell membranes. The penetration assay was used to test whether 24HC prevents the release of virus particles into the cytoplasm.

**Fig. 4:**
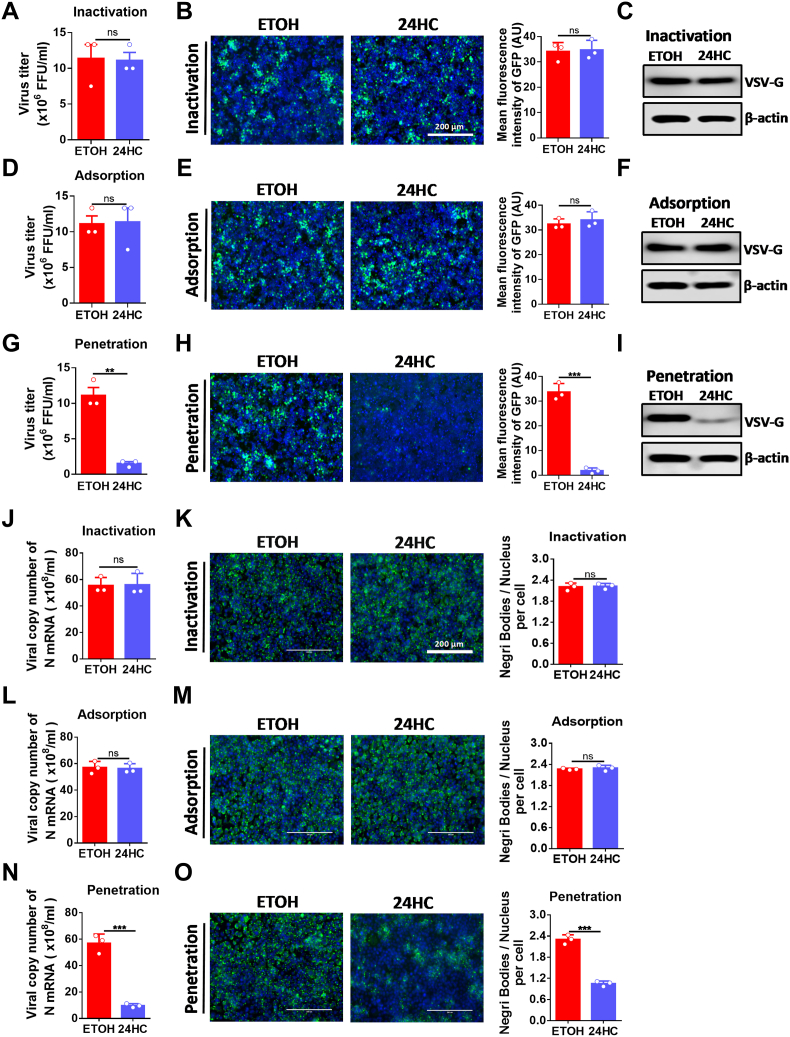
24HC exerts its antiviral function in the step of virus penetration. A-C. VSV inactivation assay. VSV-GFP (MOI = 0.01) and 24HC (5 μM) or ETOH were incubated together at 37 °C for 3 h, N2a cells cultured in 24-well plates were prechilled at 4 °C for 1 h, then the media were replaced by the mixture of 24HC or ETOH and VSV-GFP. After incubation at 4 °C for another 2 h, cells were washed and incubated at 37 °C for 12 h in a medium devoid of 24HC, and cell supernatants were collected for virus titration (n = 3) (A). Cells were fixed for detection of GFP fluorescence signals by fluorescence microscope. Mean fluorescence intensities of GFP were calculated by ImageJ software and were placed on the right side of corresponding fluorescence images (n = 3) (B), scale bar = 200 μm. Cells under the same treatment were collected for WB analysis (C). D-F. VSV adsorption assay. N2a cells cultured in 24-well plates were prechilled at 4 °C for 1 h, and then the media were replaced by a mixture of 24HC (5 μM) or ETOH and VSV-GFP (MOI = 0.01). After incubation at 4 °C for another 2 h, cells were washed and incubated at 37 °C for 12 h in a medium devoid of 24HC, and cell supernatants were collected for virus titration (n = 3) (D). Cells were fixed and observed by fluorescence microscopy (n = 3) (E), scale bar = 200 μm. Cells under the same treatment were collected for WB (F). G-I. VSV penetration assay. N2a cells cultured in 24-well plates were prechilled at 4 °C for 1 h and then incubated with VSV-GFP (MOI = 0.01) for 2 h at 4 °C. The virus-containing medium was replaced with fresh medium containing 24HC (5 μM) or ETOH, and the temperature was shifted to 37 °C for 3 h. Cells were washed and incubated at 37 °C for another 9 h in a medium devoid of 24HC, and cell supernatants were collected for virus titration (n = 3) (G). Cells were fixed and observed by fluorescence microscopy (n = 3) (H), scale bar = 200 μm. Cells under the same treatment were collected for WB (I). J, K. RABV inactivation assay. RABV (MOI = 0.01) and 24HC (5 μM) or ETOH were incubated together at 37 °C for 3 h, N2a cells cultured in 24-well plates were prechilled at 4 °C for 1 h, then the media were replaced with the mixture of 24HC or ETOH and RABV. After incubation at 4 °C for another 2 h, the cells were washed and incubated at 37 °C. After 48 h of incubation in a medium devoid of 24HC, cells were harvested to detect RABV nucleoprotein (RABV-N) mRNA levels by qPCR (n = 3) (J), or fixed and stained with anti-RABV N antibody to detect Negri bodies (RABV viral factories) and nucleus (n = 3) (K). The fluorescence intensity of Negri bodies/Nucleus was calculated using ImageJ software and placed on the right side of the corresponding fluorescence images, scale bar = 200 μm. L, M. RABV adsorption assay. N2a cells cultured in 24-well plates were prechilled at 4 °C for 1 h, and then the media were replaced with a mixture of 24HC (5 μM) or ETOH and RABV (MOI = 0.01). After incubation at 4 °C for another 2 h, the cells were washed and incubated at 37 °C. After 48 h of incubation in a medium devoid of 24HC, cells were harvested to detect RABV Nucleoprotein mRNA levels using qPCR (n = 3) (L), or fixed and stained to detect Negri bodies and nucleus (n = 3) (M), scale bar = 200 μm. N, O. RABV penetration assay. N2a cells cultured in 24-well plates were prechilled at 4 °C for 1 h and then incubated with RABV (MOI = 0.01) at 4 °C for 2 h. The virus-containing medium was replaced with fresh medium containing 24HC (5 μM) or ETOH, and the temperature was shifted to 37 °C for 3 h. The cells were washed and incubated at 37 °C. After 48 h of incubation in a medium devoid of 24HC, cells were harvested to detect RABV nucleoprotein mRNA levels by qPCR (n = 3) (N), or fixed and stained to detect Negri bodies and nucleus (n = 3) (O), scale bar = 200 μm. Statistical analysis of comparisons between groups was performed by Student's t-test (*, P < 0.05; **, P < 0.01; ***, P < 0.001; ns, not significant). Bar graph shows mean ± SD. Western blot data are representative of at least two independent experiments.

First, we tested whether 24HC could directly inactivate virus particles. The viral titer, fluorescence assay, and Western blot results showed that there was no difference between 24HC-treated and ETOH-treated cells (Fig. 4A–C), indicating that 24HC did not directly inactivate VSV. Second, the effect of 24HC on the adsorption step of VSV infection was evaluated. As shown in Fig. 4D–F, the viral titer, fluorescence assay, and Western blot results showed that there was no difference between 24HC-treated and ETOH-treated cells, indicating that 24HC did not block the attachment of VSV to cells. Finally, the effect of 24HC on VSV penetration was examined, and the results showed that VSV protein levels were significantly decreased in 24HC-treated cells than in ETOH-treated cells (Fig. 4G–I), suggesting that 24HC may block VSV penetration. However, VSV penetration is quite fast [[14], [15], [16]]. Thus we could not formally exclude an impact of the 24HC treatment on the primary viral transcription.

To support our view that 24HC may block viral penetration into cells, we also measured and confirmed that 24HC could not inactivate RABV (Fig. 4J and K) or block the RABV attachment (Fig. 4L and M), and 24HC could block RABV penetration into cells (Fig. 4N and O), which was the same mechanism as reported for 25HC inhibition of RABV replication [53]. Taken together, these results indicate that CH24H and its production 24HC can exert antiviral capacity by blocking viral penetration into cells.

### 3.5 24HC blocks the interaction between OSBP and VAPA and induces cholesterol accumulation in multivesicular bodies or late endosomes

**Fig. 5:**
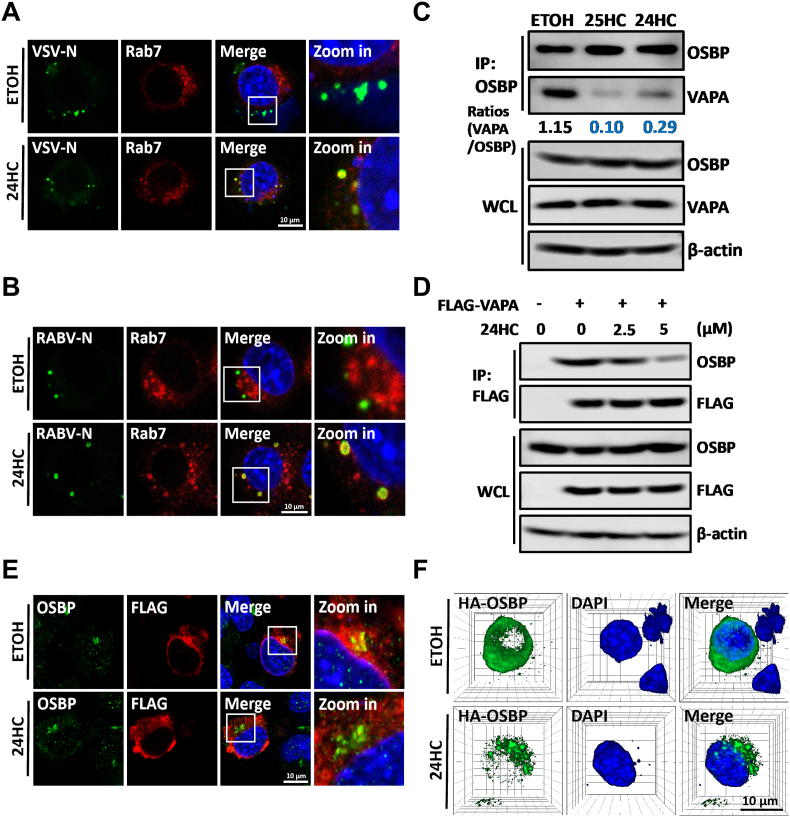
24HC sequesters the viral particles penetrating the MVB/LE and blocks the OSBP-VAPA interaction. A. N2a cells were pretreated with 5 μM 24HC for 12 h, and then infected with VSV (MOI = 10) for 3 h. Cells were analyzed for the co-localization between VSV nucleoprotein (VSV–N) and Rab7. White boxes indicate the magnified sections of the images. Scale bar, 10 μm. B. N2a cells were pretreated with 5 μM 24HC for 12 h, and then infected with RABV (MOI = 10) for 3 h. Cells were analyzed for the co-localization between RABV nucleoprotein (RABV-N) and Rab7. White boxes indicate the magnified sections of the images. Scale bar, 10 μm. C. Co-immunoprecipitation (Co-IP) was performed to detect the effect of 24HC and 25HC treatment on the interaction between OSBP and VAPA. N2a cells treated with ETOH, 24HC (5 μM), or 25HC (5 μM) for 36 h were harvested and lysed, and then pulled down using OSBP antibody as a bait. The precipitated proteins were analyzed by WB. Grey value comparison was calculated using ImageJ software. D. N2a cells were transfected with expression plasmid pCAGGS-FLAG-VAPA (named FLAG-VAPA) for 12 h and then treated with different concentrations of 24HC for 24 h. FLAG antibody was used as bait and the precipitated proteins were analyzed by WB. E. N2a cells were transfected with FLAG-VAPA for 12 h and then treated with 5 μM 24HC for 24 h, cells were fixed and stained for OSBP, FLAG-VAPA, and DAPI. The cells were then observed by confocal microscopy. White boxes indicate the magnified sections of the images. Scale bar, 10 μm. F. N2a cells were transfected with the expression plasmid pCAGGS-HA-OSBP (named HA-OSBP). After 12 h post-transfection, 5 μM 24HC was added to N2a cell culture media for 24 h. Cells were then fixed and stained with HA-OSBP and DAPI, and the Z-stack of confocal microscopy was used to acquire multi-layer images. The multi-layer images were imported into the ZEN software (version 2.3) to obtain a rendered 3D volume image. Scale bar = 10 μm. The Western blot data are representative of at least two independent experiments.

Entry of VSV and RABV particles into host cells depends on clathrin-mediated endocytosis [[14], [15], [16], [17]] and the cell endosomal pathway. Since 24HC and 25HC are similar in structure, we speculated that 24HC might function at the fusion step between the viral envelope and the endosomal membrane, as suggested by the research on 25HC [55]. We analyzed the intracellular localization of VSV and RABV at 3 h post-infection when most of the virus particles escaped from the endosomes or MVBs. Using confocal microscopy, we found that VSV nucleoprotein (VSV–N) was co-localized with Rab7, which is the marker of MVB/LE, under 24HC treatment (Fig. 5A). We also observed that RABV nucleoprotein (RABV-N) was co-localized with Rab7 under 24HC treatment (Fig. 5B). These results suggest that 24HC treatment sequesters virions in the MVB/LE.

To further explore the possible mechanism by which 24HC traps virions in the MVB/LE, a co-immunoprecipitation assay was performed to detect the interaction of OSBP with vesicle-associated membrane protein-associated protein A (VAPA) after 24HC or 25HC treatment. The result showed that the interaction between OSBP and VAPA was blocked in 24HC-treated N2a cells (Fig. 5C). Then we measured the interaction between OSBP and VAPA when different concentrations of 24HC were treated with N2a cells, the results showed that FLAG-VAPA bound less OSBP when 24HC concentration was increased (Fig. 5D). We also used confocal microscopy to observe the co-localization between endogenous OSBP and overexpressed FLAG-VAPA under the treatment of ETOH or 24HC, the results showed that 24HC treatment could decrease the co-localization efficiency between OSBP and FLAG-VAPA (Fig. 5E). In addition, we found that 24HC can cause overexpressed OSBP to form aggregated punctate structures (Fig. 5F), which was the same phenomenon observed with 25HC treatment [56], suggesting that 24HC may interfere with the interactions between OSBP and VAPA through its interaction with OSBP.

**Fig. 6:**
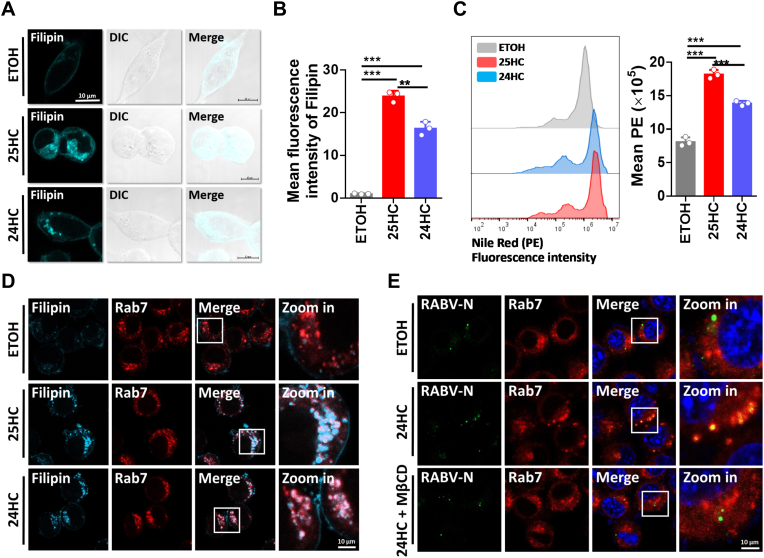
24HC induces cholesterol accumulation in multivesicular bodies or late endosomes. A. N2a cells were treated with ETOH, 24HC (5 μM), or 25HC (5 μM) for 24 h, and then fixed and stained with filipin (cyan). Images were acquired by confocal microscopy. DIC: Differential interference contrast. Scale bar, 10 μm. B. N2a cells were treated with ETOH, 24HC (5 μM), or 25HC (5 μM) for 24 h, then fixed and stained with filipin. The fluorescence signals of filipin were acquired under 360 nm excitation light, and the mean fluorescence intensities of filipin were compared (n = 3). C. N2a cells were treated with ETOH, 24HC (5 μM), or 25HC (5 μM) for 24 h, then fixed and stained with Nile red. The cells were then analyzed for PE fluorescence intensity by flow cytometry (n = 3). D. N2a cells were treated with ETOH, 24HC (5 μM), or 25HC (5 μM) for 24 h and then fixed and stained with filipin (cyan) and Rab7 (red). Cells were analyzed for the co-localization between filipin and Rab7. White boxes indicate the magnified sections of the images. Scale bar, 10 μm. E. N2a cells were pretreated with ETOH or 24HC (5 μM) for 12 h, and then MβCD (2 mM) was added. After 1 h incubation, the cells were infected with RABV (MOI = 10) for 3 h. Cells were analyzed for the co-localization between RABV nucleoprotein (RABV-N) and Rab7. White boxes indicate the magnified sections of the images. Scale bar, 10 μm. Statistical analysis of comparisons between groups was performed by Student's t-test (*, P < 0.05; **, P < 0.01; ***, P < 0.001). Bar graph shows mean ± SD. (For interpretation of the references to colour in this figure legend, the reader is referred to the Web version of this article.)

According to previous research, the interaction of OSBP and VAPA regulates cholesterol levels in LEs by transporting cholesterol from the LE to the cytoplasm [47]. Therefore, we investigated whether 24HC treatment could induce cholesterol accumulation in N2a cells. Using filipin and Nile Red staining we confirmed that both 24HC could induce cholesterol accumulation, with 25HC as a positive control (Fig. 6A–C). Then Rab7 was used as an LE marker to confirm that 24HC could increase cholesterol accumulation within LEs, and the confocal microscopy results showed that both 24HC and 25HC's treatment could lead to cholesterol accumulation of LEs (Fig. 6D). Since cholesterol-laden endosomal compartments suppress viral release from late endosomes into the cytosol by inhibiting the fusion process [47], we introduced a classical cholesterol depleting agent: methyl-β-cyclodextrin (MβCD) [57]. MβCD treatment could reduce the cholesterol concentration of late endosomes (LEs) of 24HC-treated N2a cells (Fig. S3). Then we infected N2a cells with RABV at high MOIs and showed that RABV nucleoprotein (RABV-N) colocalized with Rab7 under 24HC and MβCD treatment, whereas RABV-N did not colocalize with Rab7 under 24HC treatment (Fig. 6E). These results showed that increasing the cholesterol concentration in the LEs sequesters the viral particles penetrating the LEs, further suggesting that 24HC sequesters the viral particles that penetrate the MVB/LE by increasing the cholesterol concentration of late endosomes. Taken together, these results demonstrate that 24HC can inhibit the OSBP-VAPA interaction and cause the accumulation of cholesterol in the MVB/LE, which ultimately sequesters the viral particles penetrating the MVB/LE.

### 3.6 CH24H and 24HC inhibit the replication of multiple viruses

**Fig. 7:**
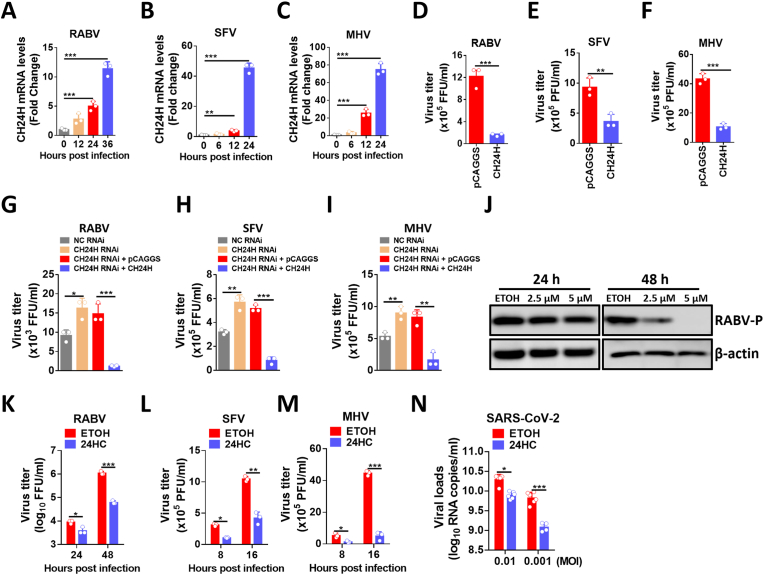
CH24H and 24HC restrict the replication of several viruses. A-C. N2a cells were infected with RABV (A) at an MOI of 0.01, SFV (B) at an MOI of 0.01, or MHV (C) at an MOI of 0.01, and cells were harvested at the indicated time points. CH24H mRNA levels were measured by qPCR (n = 3). D-F. N2a cells were transfected with pCAGGS vector or pCAGGS-CH24H for 24 h, and then cells were infected with RABV (D) at an MOI of 0.01 for 48 h, SFV (E) at an MOI of 0.01 for 16 h, or MHV (F) at an MOI of 0.01 for 16 h. The supernatants were collected for virus titration (n = 3). G-I. N2a cells were transfected with negative control siRNA (NC RNAi), CH24H-specific siRNA (CH24H RNAi), pCAGGS vector with CH24H RNAi, or CH24H with CH24H RNAi. After 24 h incubation, cells were infected with RABV (G) at an MOI of 0.01 for 24 h, SFV (H) at an MOI of 0.01 for 8 h, or MHV (I) at an MOI of 0.01 for 8 h. Cell supernatants were collected for virus titration (n = 3). J, K. N2a cells were pretreated with 24HC at the indicated concentrations (2.5 μM or 5 μM) for 12 h prior to RABV infection at MOI 0.01, the cells were harvested at 24 and 48 h post-infection. WB (J) and virus titration (K) were performed to assess viral replication levels (n = 3). L. N2a cells were pretreated with 5 μM 24HC for 12 h, and then infected with SFV at MOI 0.01 for 8 or 16 h. Cell supernatants were collected for virus titration (n = 3). M. N2a cells were pretreated with 5 μM 24HC for 12 h, and then infected with MHV at MOI 0.01 for 8 or 16 h. Cell supernatants were collected for virus titration (n = 3). N. MDCK cells were pretreated with 5 μM 24HC for 6 h, and then infected with SARS-CoV-2 at the indicated MOIs for 24 h. Cell supernatants were collected and qPCR was performed to detect SARS-CoV-2 viral load (n = 6). Statistical analysis of grouped comparisons was performed by Student's t-test (*, P < 0.05; **, P < 0.01; ***, P < 0.001). Bar graph represents mean ± SD. Western blot data are representative of at least two independent experiments.

After explaining the viral suppression mechanism of 24HC, we performed viral suppression assays of 24HC against a variety of neuroinvasive viruses. As shown in Fig. 7A–C, CH24H mRNA levels were significantly upregulated upon MHV, SFV, and RABV infection at the indicated time points. Next, N2a cells were transfected with pCAGGS-CH24H and then infected with MHV, SFV, and RABV, respectively. After incubation for the indicated periods, the cell supernatant was collected and the virus titer was calculated and the results showed that the overexpression of CH24H could cause a significant decrease in the titers of MHV, SFV, and RABV (Fig. 7D–F). To further confirm that CH24H could inhibit viral replication. NC RNAi, CH24H RNAi, CH24H RNAi with pCAGGS, and CH24H RNAi with pCAGGS-CH24H were transfected into N2a cells, respectively. N2a cells were then infected with MHV, SFV, and RABV, respectively. The cell supernatant was collected and virus titers were calculated. The results showed that transfection of CH24H knockdown can increase the virus replication level and CH24H expression can reverse this trend (Fig. 7G–I). We further overexpressed human CH24H in 293T cells and infected them with RABV. The viral titer and Western blot results showed that the replication of RABV was significantly decreased after CH24H overexpression (Figs. S4A–S4B). Together these results demonstrate that CH24H has an influence on multiple neuroinvasive virus replications.

Next, N2a cells were pretreated with 24HC and then infected with MHV, SFV, and RABV for the indicated periods. Viral titers showed that the replication level of these viruses was significantly decreased after treatment with 24HC (Fig. 7J–M). In addition, we tested the effect of 24HC on SARS-CoV-2 replication. After testing the cytotoxicity on MDCK cells treated with 24HC, we pretreated MDCK cells with 24HC and then infected them with SARS-CoV-2 at different MOIs, and the viral titer results showed that 24HC could effectively restrict SARS-CoV-2 replication (Fig. 7N). BSR and 293T cells were also pretreated with 24HC for 12 h and then infected with RABV for the indicated periods. Viral titer and Western blot results showed that the replication of RABV was significantly decreased after treatment with 24HC (Figs. S4C–S4F). Taken together, these results confirm the antiviral capacity of 24HC against multiple types of neuroinvasive viruses.

## 4 Discussion

Here we report that the expression level of CH24H can be increased upon the infection of several types of neuroinvasive viruses *in vitro*. The CH24H enzyme activity product, 24HC, can inhibit the replication of several viruses, including vesicular stomatitis, rabies virus, Semliki Forest virus, murine hepatitis virus, and SARS-CoV-2. Virus early replication cycle assays based on VSV and RABV confirmed that 24HC functions on the step of virus penetration, and further experiments showed that 24HC can block the OSBP-VAPA interaction and increase the cholesterol concentration inside MVBs/LEs, thus sequestering the virus particle from penetrating the MVBs/LEs.

Previous studies have shown that endosomal cholesterol homeostasis is linked to the interaction between oxysterols and OSBP [58]. The N-terminal pleckstrin homology domains, together with two phenylalanines and phosphatidylinositol phosphates of OSBP, form the acidic tract motif, which is responsible for the interaction of OSBP with VAPA on the surface of the endoplasmic reticulum (ER) [59,60], which is responsible for the transport of de novo synthesized cholesterol from the ER to other organelles. A recent study by Andrea Civra and colleagues showed that 25HC and 27HC could exert their antiviral capacity by inducing substantial cholesterol accumulation in the LE by preventing the interaction between OSBP and VAPA [55]. There is another important study published by Samad Amini-Bavil-Olyaee and colleagues who proposed a novel antiviral mechanism that IFITM3 could block viral entry by disrupting intracellular cholesterol homeostasis. IFITM3 can interact with VAPA to prevent VAPA-OSBP association, and then induce cholesterol accumulation in MVBs/LEs, and finally inhibit fusion of the virus particle with MVBs/LEs membranes and prevent virus entry from MVBs/LEs membranes into the cytosol [47].

Several drugs including itraconazole and fluoxetine, have been reported to cause cholesterol accumulation and have antiviral effects. Itraconazole and fluoxetine could effectively impair SARS-CoV-2 infection both *in vitro & vivo* [[61], [62], [63], [64]]. Itraconazole inhibits influenza virus *in vitro & vivo* [65,66]. Itraconazole and fluoxetine induced endolysosomal cholesterol imbalance and impaired Ebola virus infection *in vitro* [67]. 25HC treatment of mice infected with SARS-CoV-2 significantly reduced virus numbers in the lungs [68]. Since 24HC and 25HC are non-toxic natural products, 24HC may also have potential curative applications for emerging viral infections, particularly neuroinvasive viral infections.

In the present study, we show that both 24HC and 25HC can disrupt the VAPA-OSBP association and have the ability to induce cholesterol accumulation in LEs. Interestingly, during our experiments, we found that although the VAPA-OSBP interaction could be disrupted by 24HC, the treatment of 25HC at the same concentration of 24HC showed a stronger disruption of the VAPA-OSBP interaction (Fig. 5C). And the filipin staining assay showed that the cholesterol accumulation caused by 25HC is stronger than that caused by 24HC (Fig. 6B). This intriguing difference may partly explain why 25HC is more potent than 24HC in inhibiting the virus (Fig. 3J). The exact mechanism by which 24HC blocks the interaction between OSBP and VAPA was not elucidated in this article. However, 25HC has been found to interact with OSBP and it has been reported that the addition of 25HC causes OSBP to form aggregated punctate structures [56]. Our experimental data have shown that the addition of 24HC also causes OSBP to form aggregated punctate structures (Fig. 5F), so it is possible that 24HC could interfere with the interactions between OSBP and VAPA through its interactions with OSBP. Nevertheless, further crystal structural experiments are needed in our future studies.

We now know that 24HC has an antiviral capacity, which is related to 24HC interfering with the VAPA-OSBP interaction and then inducing cholesterol accumulation in MVBs/LEs. However, unlike the RABV transport in the endosomal pathway that requires both early endosomes and late endosomes [69,70]. The VSV transport in the endosomal pathway involves two distinct mechanisms: (i): Fusion of the VSV envelope and release of the VSV RNP all occur in early endosomes [16]. (ii): VSV envelope fuses with the intraluminal vesicle (ILV) within the MVB and then the membrane of the ILV fuses with the limiting MVB membrane, releasing RNP into the cytosol [71,72]. Thus, the mechanism of antiviral activity of 24HC can only partially be explained by its accumulation of cholesterol in MVBs/LEs, and it cannot be excluded that the accumulation of VSV-N in the MVB we observed (Fig. 5A) is due to the fact that 24HC inhibits viral fusion in the early endosome. It has been reported that 25HC exerts anti-coronavirus activity by depleting membrane cholesterol or inhibiting spike protein-catalyzed late endosome membrane fusion [73,74]. Given the similarity of 24HC and 25HC, it is possible that 24HC may exert its antiviral activity through different mechanisms and thus requires further investigation.

In conclusion, our data provide the first evidence that CH24H, mostly enriched in the brain, can limit neuroinvasive viral replication. More importantly, its metabolic product 24HC also possesses antiviral capacity by disrupting intracellular cholesterol homeostasis. As a natural product in the mammalian brain, 24HC may change the current understanding of the antiviral mechanism in the brain and provide new perspectives for curing patients with neuroinvasive viral infections.

## Declaration of competing interest

All the authors declare that there are no conflicts of interest.

## Data Availability

Data will be made available on request.
