# Supplementary material for: The CH24H metabolite, 24HC, blocks viral entry by disrupting intracellular cholesterol homeostasis
Source: Redox Biol. 2023 May 31;64:102769. doi: 10.1016/j.redox.2023.102769 (PMC10265526; doi:10.1016/j.redox.2023.102769)
Supplement: Multimedia component 1 [file mmc1.docx]

**Supplementary Figures for**

**The CH24H metabolite, 24HC, blocks viral entry by disrupting intracellular cholesterol homeostasis**

Yueming Yuan^1,3#^, An Fang^1,3#^, Zongmei Wang^1,3^, Zhihui Wang^1,3^, Baokun Sui^1,3^, Yunkai Zhu^4^, Yuan Zhang^1,3^, Caiqian Wang^1,3^, Rong Zhang^4^, Ming Zhou^1,3^, Huanchun Chen ^1,2,3^, Zhen F. Fu^1,3^, Ling Zhao^*1,2,3^

^1^ State Key Laboratory of Agricultural Microbiology, Huazhong Agricultural University, Wuhan 430070, China

^2^ Hubei Hongshan Laboratory, Wuhan 430070, China

^3^ Key Laboratory of Preventive Veterinary Medicine of Hubei Province, College of Veterinary Medicine, Huazhong Agricultural University, Wuhan 430070, China

^4^ School of Basic Medical Sciences, Fudan University, Shanghai 200433, China

# Yueming Yuan and An Fang contributed equally to this work.

*Corresponding author: State Key Laboratory of Agricultural Microbiology, Huazhong Agricultural University, Wuhan, 430070, China. E-mail: [zling604@outlook.com](mailto:zling604@outlook.com), lingzhao@mail.hzau.edu.cn

**Supplemental Figures**

**
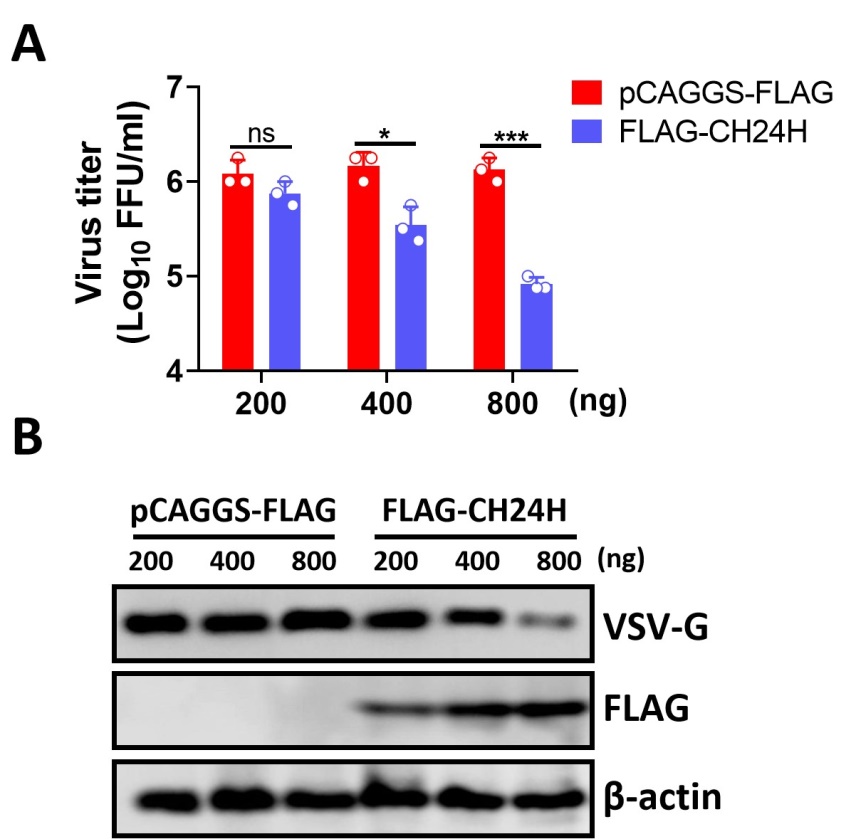
**

**Fig. S1 CH24H restrains VSV infection in 293T cells.**

A. 293T cells were transfected with the expression vector pCAGGS-FLAG or pCAGGS-FLAG-CH24H (FLAG-CH24H) at the indicated volumes. The cells were infected with VSV at an MOI of 0.01 at 24 h post-transfection. After 16 h of incubation, the supernatants were harvested for VSV titration (n=3).

B. 293T cells were transfected and infected as in (A), the cells were harvested to measure the protein levels of VSV-G, FLAG-CH24H, and β-actin by Western blotting (WB).


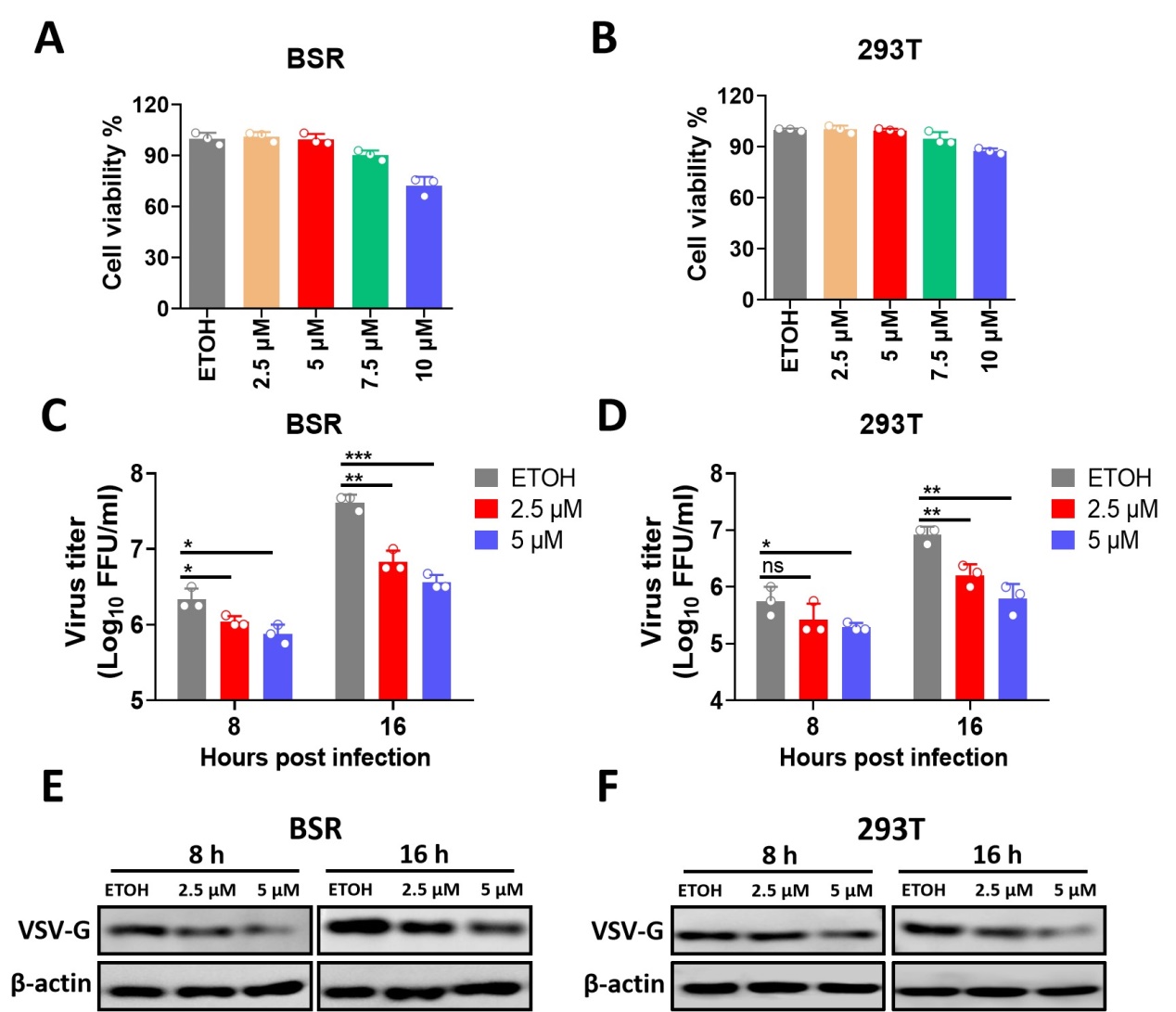


**Fig. S2 24HC inhibits VSV replication in BSR and 293T cells.**

A, B. BSR (A) and 293T (B) cells were treated with different concentrations of 24HC or ethanol (ETOH) for 36 h, then the supernatants were discarded and the cytotoxicity was measured (n=3).

C, D. BSR (C) and 293T (D) cells were pretreated with 24HC at the indicated concentrations (2.5 µM or 5 µM) for 12 h prior to VSV infection at MOI 0.01, after the indicated incubation times, the supernatants were harvested for VSV titration (n=3).

E, F. BSR (E) and 293T (F) cells were treated and infected as in (C, D), the cells were harvested to measure the protein levels of VSV-G and β-actin by WB.


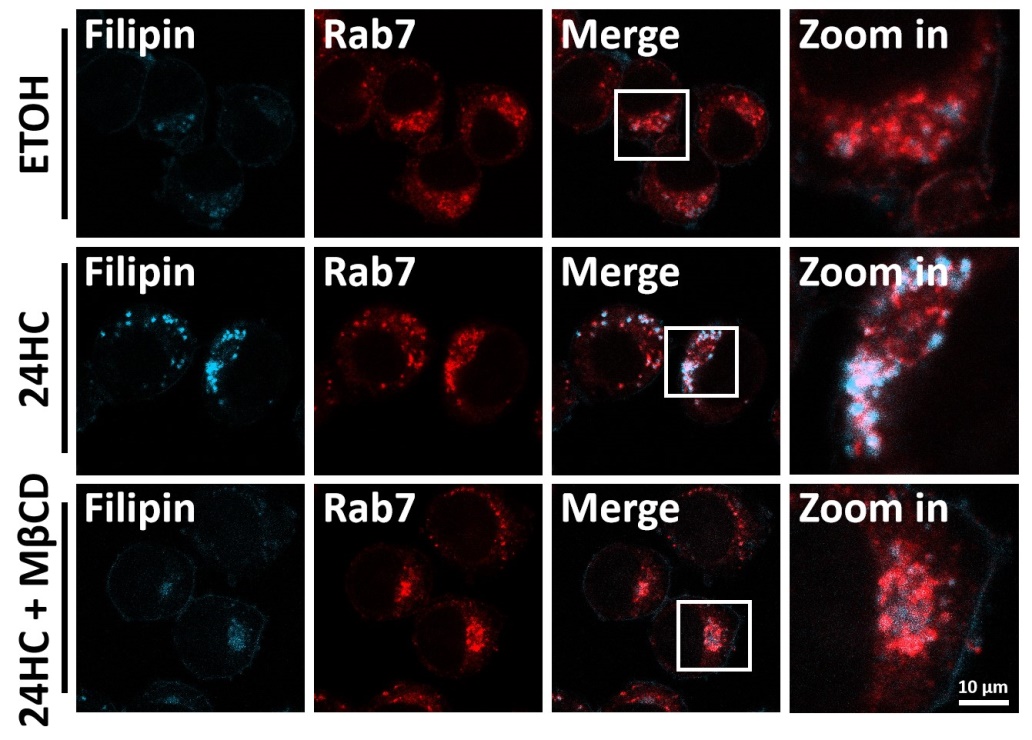


**Fig. S3 MβCD treatment reduces the cholesterol concentration of late endosomes.**

N2a cells were pretreated with ETOH or 24HC (5 µM) for 12 h, and then MβCD (2 mM) was added. After 4 h incubation, cells were fixed and stained with filipin (cyan) and Rab7 (red). White boxes indicate the magnified sections of the images. Scale bar = 10 μm.


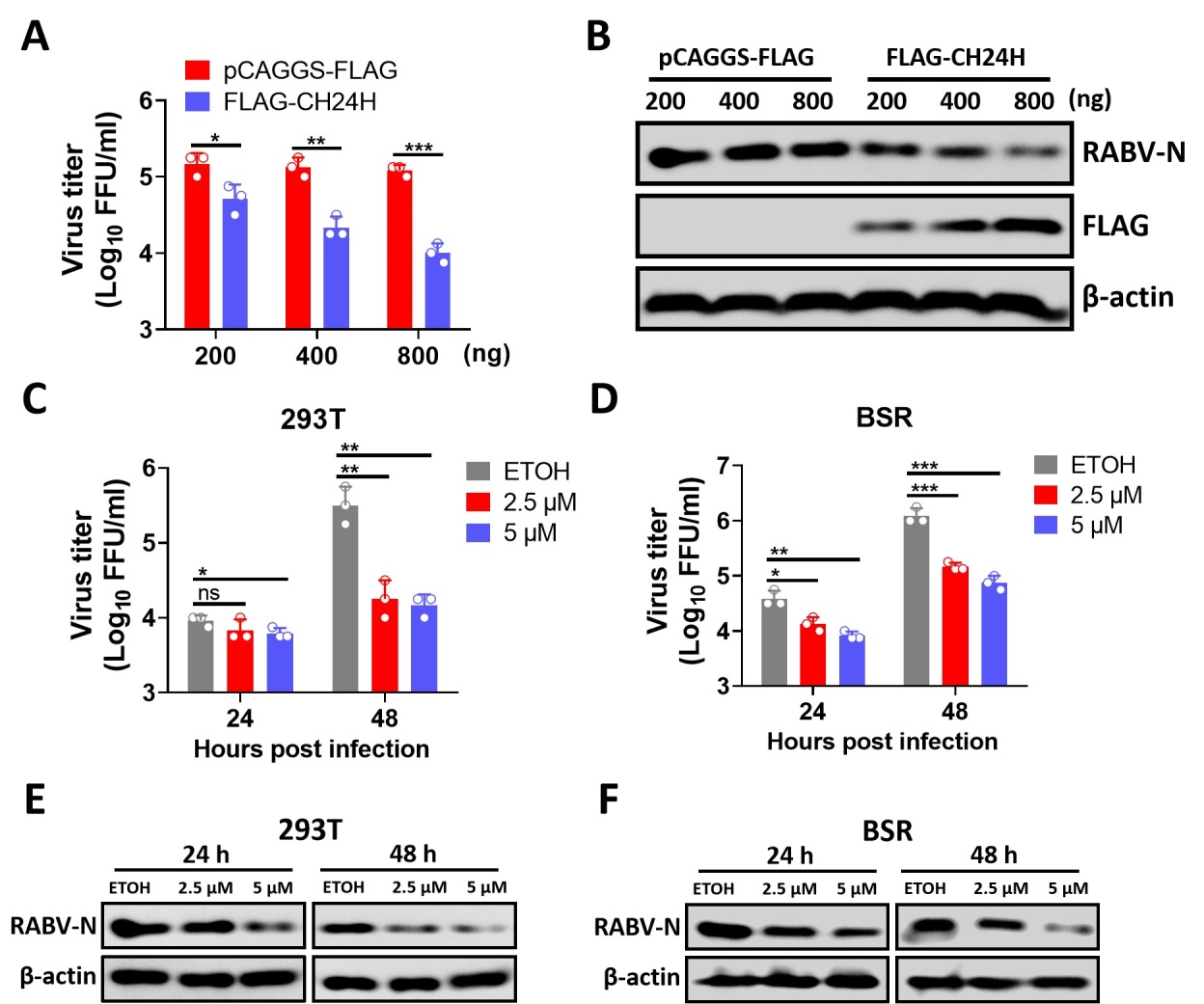


**Fig. S4 CH24H and 24HC inhibit RABV replication in BSR and 293T cells.**

A. 293T cells were transfected with the expression vector pCAGGS-FLAG or pCAGGS-FLAG-CH24H (FLAG-CH24H) at the indicated volumes. The cells were infected with RABV at an MOI of 0.01 at 24 h post-transfection. After 48 h of incubation, the supernatants were harvested for RABV titration (n=3).

B. 293T cells were transfected and infected as in (A), the cells were harvested to measure the protein levels of RABV-N, FALG-CH24H, and β-actin by WB.

C, D. 293T (C) and BSR (D) cells were pretreated with 24HC at the indicated concentrations (2.5 µM or 5 µM) for 12 h prior to RABV infection at MOI 0.01, after the indicated incubation times, the supernatants were harvested for RABV titration (n=3).

E, F. 293T (E) and BSR (F) cells were treated and infected as in (C, D), the cells were harvested to measure the protein levels of RABV-N and β-actin by WB.
